# Training satisfaction and future employment consideration among physician and nursing trainees at rural Veterans Affairs facilities in the United States during COVID-19: a time-series before and after study

**DOI:** 10.3352/jeehp.2024.21.25

**Published:** 2024-09-24

**Authors:** Heather Northcraft, Tiffany Radcliff, Anne Reid Griffin, Jia Bai, Aram Dobalian

**Affiliations:** 1 Veterans Emergency Management Evaluation Center, US Department of Veterans Affairs, Los Angeles, CA, USA; 2 School of Public Health, Texas A&M University, College Station, TX, USA; 3 College of Public Health, The Ohio State University, Columbus, OH, USA; Hallym University, Korea

**Keywords:** COVID-19, Logistic models, Physicians, Rural population, United States

## Abstract

**Purpose:**

The coronavirus disease 2019 (COVID-19) pandemic limited healthcare professional education and training opportunities in rural communities. Because the US Department of Veterans Affairs (VA) has robust programs to train clinicians in the United States, this study examined VA trainee perspectives regarding pandemic-related training in rural and urban areas and interest in future employment with the VA.

**Methods:**

Survey responses were collected nationally from VA physicians and nursing trainees before and after COVID-19 (2018 to 2021). Logistic regression models were used to test the association between pandemic timing (pre-pandemic or pandemic), trainee program (physician or nurse), and the interaction of trainee pandemic timing and program on VA trainee satisfaction and trainee likelihood to consider future VA employment in rural and urban areas.

**Results:**

While physician trainees at urban facilities reported decreases in overall training satisfaction and corresponding decreases in the likelihood of considering future VA employment from pre-pandemic to pandemic, rural physician trainees showed no changes in either outcome. In contrast, while nursing trainees at both urban and rural sites had decreases in training satisfaction associated with the pandemic, there was no corresponding effect on the likelihood of future employment by nurses at either urban or rural VA sites.

**Conclusion:**

The study’s findings suggest differences in the training experiences of physicians and nurses at rural sites, as well as between physician trainees at urban and rural sites. Understanding these nuances can inform the development of targeted approaches to address the ongoing provider shortages that rural communities in the United States are facing.

## Graphical abstract

[Fig f4-jeehp-21-25]

## Introduction

### Background/rationale

Rural populations confront multiple barriers to accessing health care, which are recognized by designations as Health Professional Shortage Areas (HPSAs), medically underserved areas, or medically underserved populations based on limited numbers of health professionals available or employed locally. The inability to recruit or retain healthcare professionals in rural areas may be tied to more limited exposure to training opportunities by future healthcare professionals in rural communities; indeed, research suggests that training programs with specific rural training experience are successful in leading graduates to practice in rural areas subsequently [1]. As the largest health professional training system in the United States [2], the US Department of Veterans Affairs (VA) has established training programs that seek to improve access to healthcare for the 5 million veterans living in rural areas [3-5]. For example, the VA’s Office of Rural Health developed a training initiative to increase clinical training opportunities, adding trained providers to rural facilities and leading to better care for rural veterans [3]. Another strategy to increase the supply of rural healthcare professionals within the VA has been the utilization of international medical graduates (IMGs), who are more likely to practice in rural areas compared to US graduates [6,7]. The employment of IMGs in rural communities continued during the coronavirus disease 2019 (COVID-19) pandemic [6].

The pandemic exacerbated previous obstacles in rural healthcare settings [7] and created new challenges for rural communities as many rural health professions training programs experienced difficulty finding preceptors and supervisors. Training was delayed or eliminated at some locations [8-10]. Although the VA funded programs to increase telehealth utilization for rural veterans even before COVID-19 [3,5], telehealth use exponentially increased after the pandemic began, including in rural communities [5]. Research has demonstrated the importance of telehealth training for healthcare professional trainees during COVID-19, both in terms of the provision of telehealth services as well as the availability of virtual precepting and supervision for trainees [1,8-10]. While research describing the changes to health professions training at rural locations during COVID-19 focused on satisfaction with telemedicine and education regarding virtual care [1,9], research related to training satisfaction and intention to leave their training program is lacking.

### Objectives

This study investigated whether and how the COVID-19 pandemic impacted learning experiences for health professions trainees, including physician and nurse trainees, in rural and urban VA facilities, specifically using national survey data from pre-pandemic and post-pandemic onset to identify trainees’ satisfaction with their VA training experiences and their likelihood to consider future VA employment. Physician and nurse trainees are the 2 largest training groups at the VA. Furthermore, the VA provides extensive healthcare professional education and training across the United States and utilizes IMGs, particularly in rural communities [6]. As such, changes in trainee satisfaction and related impacts on future VA provider employment can have national and international importance. Further, this analysis can inform how training programs improve trainee satisfaction and mitigate the impact of future disruptions on rural training programs and subsequent employment of rural health professionals.

## Methods

### Ethics statement

The Department of Veterans Affairs, Greater Los Angeles Institutional Review Board (1767580-1), approved this study as a quality improvement project. The VA’s Office of Academic Affiliations (OAA) assures all respondents of their anonymity at the time of data collection. OAA collects all data used in the study as part of its routine improvement efforts, and participation in the survey is voluntary.

### Study design

This is a time-series before-and-after study. It was described according to the TREND (Transparent Reporting of Evaluations with Nonrandomized Designs) statement available at https://www.cdc.gov/trendstatement/index.html.

### Setting

OAA oversees the education and training of health professionals within the VA. Most trainees complete a one-year rotation at a VA facility. At the rotation’s conclusion, they are emailed a link to complete the Trainee Satisfaction Survey (TSS) via an online survey. Trainees are asked to complete the TSS only once during the academic year. Responses for the current analyses of trainees were collected across 155 VA facilities for 3 academic years from August 3, 2018, to July 30, 2021.

### Participants

Trainees were identified as individuals who completed at least 1 rotation at a VA facility during the referenced timeframe. Only trainees who indicated a physician or nursing program were included in the analysis. Medical students were removed from the sample because their experiences and earlier stage of education would markedly differ from those of the other groups (i.e., residents and fellows).

### Interventions

The period before the pandemic (“pre-pandemic”) was defined as any response provided before February 29, 2020, while the COVID-19 pandemic period (“pandemic”) included any response after April 1, 2020. Data from March 2020 were excluded because the TSS did not indicate whether the training period occurred before or after the COVID-19 public health emergency was declared.

### Outcomes

Responses to the question “Overall, how satisfied are you with your VA training experience?” were used to measure overall satisfaction and were collapsed into positive and negative response groups: Satisfied (satisfied/very satisfied) and Dissatisfied (dissatisfied/very dissatisfied). Responses to the question “As a result of your training experience, how likely would you be to consider a future employment opportunity at a VA medical facility?” were used to measure the likelihood of future VA employment and were collapsed into a positive and negative response group: Likely (likely/very likely) and Unlikely (unlikely/very unlikely). The TSS does not include a “neutral” response option.

### Data sources/measurement

As mentioned above, OAA encourages trainees from all VA trainee facilities to complete the TSS every year. Survey items included satisfaction with different components of the VA training experience, likelihood to consider future VA employment, and demographic data (e.g., training program, specialty, and facility). This study defined the rurality of VA sites using the rural-urban commuting area system [4]. Urban medical centers were defined as being located within census tracts, with at least 30% of the population residing within an urbanized area. Rural medical centers were located within census tracts, with less than 30% of the population commuting to an urbanized area and more than 10% commuting to any community larger than an urbanized cluster.

### Bias

Our data set did not allow us to determine whether trainees were IMGs. Because IMGs are more common in rural communities, it is possible that at least some of our findings might relate to differences in satisfaction and interest in future employment between IMGs and domestic medical graduates.

### Study size

Using Stata SE ver. 17.0 (Stata Corp.), margin commands were used to calculate 95% confidence intervals (CIs) for both outcome variables.

### Assignment method

Individual trainees were separated into groups based on when their rotation was completed (pre-pandemic or pandemic; see “Interventions” section) and based on their professional training program (physician or nursing).

### Unit of analysis

As mentioned above, individual trainees were assigned to groups based on when their TSS was completed (pre-pandemic versus pandemic) and which training program was indicated on the survey (physician versus nurse).

### Statistical methods

Logistic regressions were conducted using Stata SE ver. 17.0 (Stata Corp.) to test the association between pandemic timeline (pre-pandemic or pandemic), training type (physician or nurse), and a pandemic group-by-training group interaction term for the 2 outcome variables: overall satisfaction and likelihood of future VA employment. Stata’s “margin” commands were used to calculate the probability of reporting satisfied/very satisfied and likely/very likely for each group. These analyses were completed separately for urban and rural medical center sites to identify if geographic location was associated with training satisfaction and future employment plans.

## Results

### Participants

**Fig. 1.**
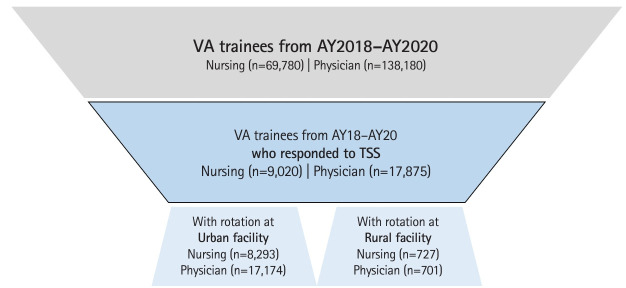
Sample of nursing and physician Veterans Affairs (VA) trainees who responded to Trainee Satisfaction Survey (TSS). AY, academic year.

For academic years 2018–2019, 2019–2020, and 2020–2021, 203,571 physician and nursing trainees received training at a VA facility [2]. Across these 3 academic years, 26,895 trainees responded to the TSS (13% response rate), including both physician (n=17,875) and nursing (n=9,020) trainees (Fig. 1).

### Overall satisfaction

**Fig. 2.**
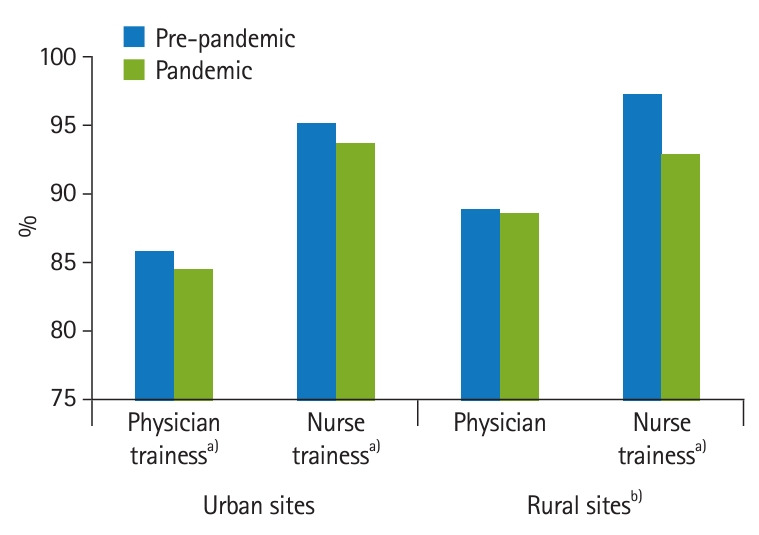
Percent of Veterans Affairs (VA) physicians and nurses trainees who were satisfied/highly satisfied with VA training before and after coronavirus disease 2019 (COVID-19) pandemic. ^a)^Pandemic group difference P<0.05. ^b)^Pandemic-by-training interaction P<0.05.

Across all periods and training locations, more than 80% of physician trainee respondents and more than 90% of nurse trainee respondents in the sample reported they were satisfied or highly satisfied with their VA training experience. The pandemic timeline by training program interaction was insignificant for urban sites. Overall training satisfaction from pre-pandemic to pandemic periods decreased for both urban physician trainees (85.85% versus 84.45%; 95% CI, -2.49 to -0.31; P=0.012) and urban nursing trainees (95.21% versus 93.69%; 95% CI, -2.52 to -0.52; P=0.003). In contrast to the urban sites, there was a significant pandemic timeline by training program interaction at rural sites (P=0.030). While physician trainees showed no statistically significant change in overall training satisfaction after the pandemic began (88.83% versus 88.62%; 95% CI, -4.89 to 4.48; P=0.932), nursing trainees reported a statistically significant decrease in overall VA training program satisfaction (97.19% versus 92.86%; 95% CI, -7.53 to -1.12; P=0.008) (Fig. 2, Table 1).

### Likelihood of future employment

**Fig. 3.**
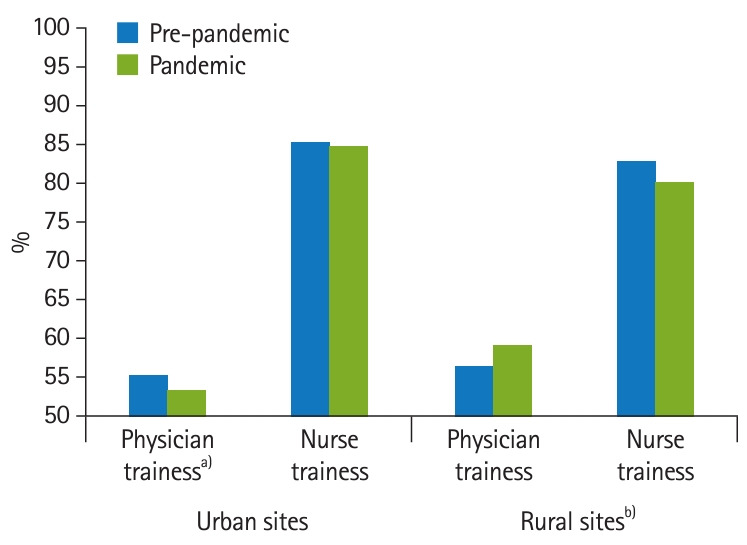
Percent of Veterans Affairs (VA) physicians and nurses trainees who were likely/very likely to consider future VA employment before and after coronavirus disease 2019 (COVID-19) pandemic. ^a)^Pandemic group difference P<0.05. ^b)^Pandemic-by-training interaction P<0.05.

Across all periods and training locations, less than 60% of physician trainee respondents indicated they were likely to seek out VA as a future employment opportunity; while, more than 75% of nurse trainees were open to future VA employment. There was no significant pandemic group-by-training program group interaction for the likelihood of future employment for either urban or rural sites; however, there was a notable group difference. Physician trainees at urban sites reported a significant decrease in willingness to consider future VA employment from pre-pandemic to pandemic (55.26% versus 53.24%; 95% CI, -3.55 to -0.49; P=0.010) whereas there was no significant difference for nursing trainees (85.26% versus 84.65%; 95% CI, -2.17 to 0.94; P=0.440). Neither physicians nor nursing trainees at rural sites showed any differences from pre-pandemic to pandemic (Fig. 3, Table 1).

## Discussion

### Key results

While physician trainees at urban sites reported decreases in training satisfaction and corresponding decreases in the likelihood of considering future VA employment from pre-pandemic to pandemic, physicians at rural sites showed no statistically significant changes in either outcome associated with COVID-19. In contrast, nursing trainees at urban and rural sites reported decreased overall training satisfaction associated with the pandemic. However, there was no corresponding difference in future employment interest for nurse trainees at urban or rural sites. However, nurse trainees had higher overall satisfaction across both training site locations and periods and a higher reported likelihood to consider VA future employment compared to physician trainees.

### Interpretation

Our study adds to the literature by suggesting that rural communities and facilities may have certain factors that mitigate against some of the potential negative consequences of COVID-19 on healthcare professionals and trainees [11]. We found VA physician trainees in rural communities did not report decreased satisfaction or a lower likelihood of seeking future VA employment compared to trainees in urban settings. In contrast, urban and rural nursing trainees reported decreased satisfaction, although their likelihood to consider future VA employment was not impacted for either group. Future studies are needed to investigate differences in staffing stability and rural resiliency in attracting new providers to identify whether these findings are unique to VA trainees.

### Comparison with previous studies

Previous research demonstrated the pandemic’s association with declines in healthcare profession training and education especially in rural areas [8-10]. Prior work has shown that the pandemic exacerbated shortages in healthcare professionals for rural populations [8]. Nonetheless, previous literature has also indicated protective factors for providers at rural locations [11]. One study found that during COVID-19, providers at rural sites had less burnout and more compassion satisfaction than their urban counterparts; however, this study included practicing providers and other hospital staff and did not include trainees [11]. Research regarding the differences between urban and rural clinicians during the pandemic is mixed, with some research demonstrating protective factors [11] and other research identifying more negative impacts at rural sites (e.g., harassment because of their work and intent to leave due to the pandemic) [12]. These mixed outcomes may relate to differences in career status (practicing provider or trainee) and profession (physician or nurse) [11,12].

### Limitations

The current study has limitations. The TSS assessed self-reported likelihood to consider future VA employment. We do not know whether the trainees sought VA employment. Another limitation is the historically low response rate of the TSS (11%–14% in recent years before and during COVID-19), in addition to the limited number of rural VA sites compared to their urban counterparts. Finally, while we cannot determine the exact dates of a trainee’s rotation at VA, OAA does request that trainees take the survey upon completion of their rotation.

### Generalizability

Research indicates up to 26% of residents enrolled in an accredited residency program were IMGs [7]. Within the VA, IMGs are often used in HPSAs to meet the demand of medical providers, and this practice has continued throughout the COVID-19 pandemic [6,7]. This phenomenon is not limited to the United States, as other countries recruit IMGs for remote areas [7]. As the VA continues to utilize IMGs for rural areas, it is important to consider opportunities to improve both IMG and non-IMG training experiences for nurses and expand the VA’s approach to training physicians in rural communities.

### Suggestions

This study does not include data to identify reasons for the discrepant findings between physicians and nursing trainees at rural sites, but we offer some potential explanations for further study. Perhaps nurse trainees in rural communities experienced differential stressors on their satisfaction during the pandemic compared with their physician counterparts, although not to the extent that they were willing to alter their calling to become nursing professionals; this possibility was not explicitly evident in the literature and should be considered in future studies. Compared with urban facilities, rural training sites may be better structured to enhance or maintain satisfaction among physicians compared to nurse trainees. One study of nurse practitioner residents at rural clinics found trainee attrition because the training program did not offer the anticipated experience [13]. It is also important to acknowledge the differences in training models between nurse trainees who, even before the pandemic, may have struggled to obtain preceptors and may have been required to identify their clinical preceptors [14,15]. In contrast, physician trainees can rely on the mentorship of an established and funded residency program. The pandemic only increased nurse trainees’ difficulties finding required preceptors, including in rural populations [11]. It is also possible that the mentorship received by rural nurse trainees was more impacted than that of physicians, perhaps because of pandemic-related differential workload impacts between rural nurses and physicians. The relatively larger decline in satisfaction among rural nurse trainees compared to their urban counterparts suggests the possibility that nurse trainees in rural areas experienced a relatively larger change in their training circumstances during COVID-19 compared to before the pandemic. Previous literature in a sample of local health department employees (with many rural departments often staffed by nurses) has shown that compared to urban staff; rural staff were more likely to report their reason for leaving was due to stress and burnout; the study also found proportionally more rural staff reporting wanting to leave work because of COVID-19 and overload or burnout among those who intended to leave their jobs [12]. The results of the current study may also shed light on potential mitigating factors (e.g., well-established mentorship programs) to health profession training during future disasters. Future studies with more recent data would also help determine the longer-lasting impacts of the pandemic on health professional training.

### Conclusion

VA has a robust training system for nurses and physicians that may have mitigated some of the negative healthcare workforce impacts of COVID-19. Results indicate there may be potential approaches to improve training for healthcare professionals and broaden the appeal of VA as a future employment option. The above results also suggest improving training experiences for nurses and expanding the VA’s approach to training physicians in rural communities. The above results urged us to identify what those approaches might be.

## Figures and Tables

**Figure f4-jeehp-21-25:**
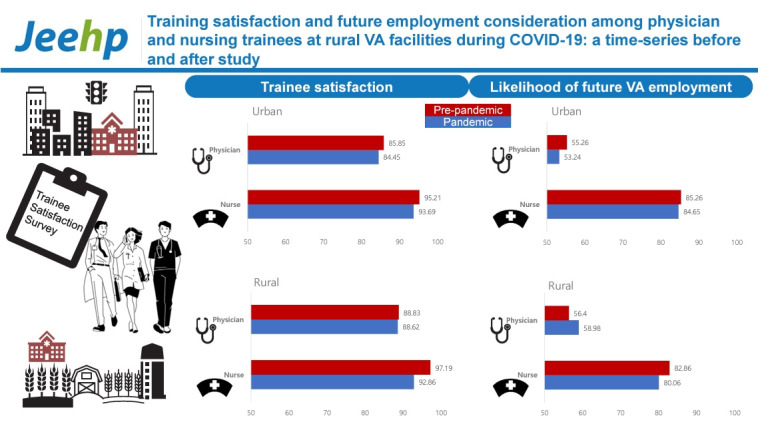


**Table 1.** Logistic regression predicting overall satisfaction and likelihood of trainees considering future Veterans Affairs employment by group

	% Satisfied	P-value	Pre-pandemic vs. pandemic difference in % satisfied (95% CI)	% Likely	P-value	Pre-pandemic vs. pandemic difference in % likely (95% CI)
Urban						
Physician		0.012			0.010	
Pre-pandemic	85.85		NA	55.26		NA
Pandemic	84.45		-1.40 (-2.49 to -0.31)	53.24		-2.02 (-3.55 to -0.49)
Nurse		0.003			0.440	
Pre-pandemic	95.21		NA	85.26		NA
Pandemic	93.69		-1.52 (-2.52 to -0.52)	84.65		-0.61 (-2.17 to 0.94)
Rural						
Physician		0.932			0.490	
Pre-pandemic	88.83		NA	56.40		NA
Pandemic	88.62		-0.21 (-4.89 to 4.48)	58.98		2.58 (-4.74 to 9.90)
Nurse		0.008			0.333	
Pre-pandemic	97.19		NA	82.86		NA
Pandemic	92.86		-4.33 (-7.53 to -1.12)	80.06		-2.80 (-8.48 to 2.87)

CI, confidence interval; NA, not applicable.

## Supplementary materials

Supplement 1. Audio recording of the abstract.
